# Determining the Ability of Senior Dental Students to Detect Interproximal Caries Using Different Radiographic Techniques

**DOI:** 10.1155/2024/9877819

**Published:** 2024-10-01

**Authors:** Muzan Abdalla, Saadika B. Khan

**Affiliations:** Department of Prosthodontics University of the Western Cape, Cape Town, South Africa

**Keywords:** accuracy in presence and size of caries lesion, bitewings, diagnostic techniques, digital imaging, interproximal caries, printed film, undergraduate students

## Abstract

**Objective:** To evaluate the diagnostic capability of 5th-year students using digital imaging, conventional bitewing (BW) radiographs, and printed film on paper to detect interproximal caries lesions.

**Methods:** A cross-sectional study was conducted with senior dental students. Three different radiographs: digital, BW, and printed films on paper were used; thus, nine radiographs, each with a 2-min viewing time, were considered by students along with a questionnaire. A control group of specialists from Prosthodontics and Radiology had finalized the answers prior to conducting the study. The appropriate responses were divided into five categories: R0: Intact surface, R1: Radiolucency in the outer half of enamel, R2: Radiolucency in the inner half of enamel, R3: Radiolucency in the outer half of dentin, and R4: Radiolucency in the inner half of dentin. Students' responses were analyzed using a one-way analysis of variance (ANOVA) test and a *t*-test.

**Results:** Ethics for the study was obtained from the institutional committee (Reg No: BM19/9/8). When compared with the control group using ANOVA testing, the results showed good *detection accuracy* with a success rate of ~64 accuracy. There was a significant difference in the outcomes when detecting the presence of the caries lesion between the three diagnostic techniques (*p*  > 0.001). In detecting the *size* of the carious lesion, the students' ability was recorded as poor. The highest average for detecting the presence of the carious lesion was correspondent to the printed film on paper method.

**Conclusions:** Senior dental students have shown good accuracy in detecting the presence but not the size of interproximal caries on all radiographs viewed.

**Clinical Significance**: Teachings and availability of diverse radiological diagnostic techniques ensured a reasonable level of understanding and use of the diagnostic caries risk assessment methodologies as required in restorative treatment planning.

## 1. Introduction

Dental caries is one of the most common chronic noncommunicable diseases affecting the oral cavity and it is caused by an imbalance in the oral microbial community known as dental biofilm [1–3]. Several diagnostic phases are included to detect and confirm the presence of caries. Traditionally, relying on visual examination for caries detection with or without tactile sensation is very common. In order to evaluate the carious state of the teeth of a patient, we decide whether caries is absent or present based on obvious signs such as color changes, hardness, and translucency using explorers and light [4]. This visual detection is usually supported and confirmed by bitewing (BW) radiography during the diagnostic stage clinically [4].

Dental practitioners' understanding and knowledge related to the concept of dental caries has evolved, including detection, classification, and management thereof using systems such as the International Caries Detection and Assessment System [5, 6]. *Carious lesion detection* implies an objective method of determining whether or not the disease is present [6, 7]. One of the most important roles of dental practitioners is to detect caries before they progress to irreversible lesions [8].

According to a study by Safi et al. [9], 25%–42% of interproximal caries may not be seen on clinical examination without including radiographic screening methods. Even though visual inspection is regarded as the most basic and widely used caries detection method in dental clinics, it has limitations in separating the different caries phases because it can only perceive changes reflected on the surface [10, 11]. Most interproximal dental caries lesions, for example, begin below the proximal contact point, making them difficult to identify visually. Therefore, the use of radiographs, of which there are several different types, as an aid to detect these lesions early on will further enhance diagnosis and the clinician's diagnostic ability [12].

### 1.1. BW Radiography

BW radiographs are the most commonly used and found diagnostic tool to *detect* interproximal caries [13]. Detecting carious and noncarious lesions at an early stage is of great importance [14]. Depending on the size, smaller lesions respond better to either preventive or re-mineralization as a choice of treatment [15]. But there is also a cause for concern because failure in diagnosing caries early on may lead to the progression of these lesions with unnecessary extensive restorative treatment and destruction of sound teeth [14].

With BW radiographs, however, the *depth* of the caries lesion is underestimated, and these radiographs are rather more suited to detecting dentin caries rather than enamel caries [16]. These BWs are also used to examine interproximal surfaces due to the presence of neighboring teeth and gingival tissue in the cervical areas [17]. Interproximal caries progress rapidly, and these are difficult to diagnose due to the lesion's site and the proximity of other oral structures [18].

### 1.2. Digital Radiography

When radiographic images are in digital form and can be shown on a computer monitor, the phrase “digital imaging” is used, sometimes known as filmless radiography [18]. Digital radiographs are now available at many universities' dental clinics, and these are included in the teachings. Therefore, the diagnostic accuracy of detecting carious lesions has improved. Many manufacturers have created image analysis software that can indicate areas of image density disparity, which is consistent with interproximal caries, alerting practitioners to areas that require further investigation [19].

### 1.3. Near-Infrared Light Transillumination (NILT)

NILT is a radiological technique for medical imaging but can also be used for the detection of caries [20–22]. This optical technique was later modified to a digital imaging fiber-optic transillumination (FOTI) system [20–22]. It is observed to be more nonirradiative and used as an adjunctive and sensitive method for early caries detection [22]. The increased focus on the use of less ionizing radiation and treating early caries in a minimally invasive manner has driven research into the possibilities of light-based caries detection methods [23]. In addition to being safer (nonirradiative) and more accurate (sensitivity), it is also more accurate compared to BW radiography in detecting early tooth demineralization [24]. This was shown in a clinical study with a device that used NILT, particularly DIAGNOcam, that detected the enamel lesion of proximal permanent teeth more accurately than the BWs [23, 25].

### 1.4. FOTI

Transillumination is a technique that uses optic fiber technology to target a tooth with high-intensity white light from a hand-held device [26]. This procedure can be utilized on all of the patient's dental surfaces, but it is especially useful in interproximal lesions of anterior teeth, where the buccolingual enamel thickness is lower than that of posterior teeth [25–27]. FOTI techniques should not be disregarded since sufficient evidence supports its high specificity and sensitivity in caries diagnosis, including having digital variants such as the digital FOTI [28].

### 1.5. Light Emitting Diode

Another device was invented to detect occlusal and interproximal caries [29]. Its effectiveness on wet teeth has been recorded, but removal of plaques and other organic deposits (stains and porosities) is a must before the examination to prevent interruptions of the autofluorescence signal, which may record false-positive readings [19, 24].

### 1.6. Cone Beam Computed Tomography (CBCT)

A 3-dimensional radiographic modality projected on computers is used more often in dental clinics [30–32]. It is a volumetric-based image where the tomographic sections are obtained in given resolutions which then are joined together to form a cubic pixel [31–34]. Qu et al. [35] stated that CBCT is most likely to be a promising technique for analyzing previously detected small carious lesions as well as undetected caries beneath a sound surface. Whereas, Valizadeh et al. [33] reported that this technology may aid in the identification of proximal caries, but its use is recommended with caution due to the greater dosage exposure. Thus, it is still debatable whether CBCT is better than conventional methods in the detection of dental caries. Other disadvantages of CBCT that impact on its daily inclusion in dental clinics are its high radiation dose compared to plain film radiography, the presence of artifacts (metal and movement), noise production, poor soft tissue contrast and structure, and that the edges of anatomical features lose their definition and that it is too costly [30, 36].

A National Institute of Health's consensus statement and other experts indicated that placing a tooth restoration does not halt the caries process [37, 38]. It further states that the focus of clinicians should rather be on enhancing preventive and diagnostic processes. Given the importance of early diagnosis of caries, it is necessary that dental students, during their education, obtain an acceptable level of competence in the detection of caries using improved methods, prevention, formation, progression, and the appropriate management of these carious lesions [5]. Detecting caries before they proceed to irreversible lesions is one of the most important roles of dental professionals. This detection plays an important role in avoiding irrelevant restorative treatment and removal of noncarious tooth structures [14].

This study, therefore, targeted a group of final-year dental students aiming to assess their ability in detecting both the *presence and depth of interproximal caries* using different radiographic techniques.

## 2. Research Question

The research question for this study is as follows: “Are 5th-year dental students capable of detecting the presence and depth of interproximal caries lesions using various radiographic diagnostic methods?”

## 3. Methodology

For this cross-sectional study, the 5th-year Dentistry class of 2019 was the sample cohort. Patients' radiographic images were collected as study images which were used to assess the students' diagnostic abilities. These radiographs were selected using a systematic random sampling approach based on the inclusion criteria: radiographs included were collected from those taken only at the Tygerberg Oral Health Center, Cape Town; it had to be good quality radiographs showing early signs or no signs of interproximal caries with the neighboring teeth.

A control group (gold standard) consisting of specialists from both the Radiology and Prosthodontics Dentistry Departments (*N* = 4) was used to finalize the study of radiographic images, that is, BW and digital radiography and printed film on paper. The goal of the gold standard was to compare the students' results to it. The control group also included the primary researcher to finalize the inclusion of radiographs, where agreement was reached regarding the presence/absence of caries and their depth in all three different radiographic techniques. The control group (*N* = 4) examined the same radiographs, and the agreement for inclusion was made when two of the members shared the same answer. When conflicts in the answer were experienced, a third opinion was taken into consideration. The radiographs included for this study were divided into three categories for both groups (the control and student groups) before the study commenced: *intact surface* (no caries), *early-stage interproximal caries* (enamel caries), and *advanced-stage interproximal caries* (dentin with/without pulp involvement).

A questionnaire, which was composed of two sections and related to the aim of this study, included general knowledge related to diagnostic methods, and questions specific to this study were administered to the students to complete. The questions were derived from similar studies that involved students to determine their diagnostic abilities [39, 40]. It has been slightly altered to fit the aim and objectives of this current study.

The students had to detect the size of the carious lesion present radiographically given the choice of one or more of the following charting options: R0: Intact surface, R1: Radiolucency (RL) in the outer half of enamel, R2: RL in the inner half of enamel, R3: RL in the outer half of dentin, R4: RL in the inner half of dentin [41]. The test was based on three different radiographic diagnostic tools: BW radiography, digital radiography, and printed film on paper. The statistical tests that were used in this study were the analysis of variance (ANOVA) test and *p* value test (the *t*-test). ANOVA tests further explain states that there are some statistical differences between the means of three or more independent groups [42].

## 4. Result

A total number of *N* = 66 (86.8%) students from a class of 76 participated in this study after obtaining ethical approval from the institutional ethics review board (Reg No: BM 19/9/8). The responses related to the questionnaire included some initial general questions for Section 1, such as the most common diagnostic tool used, preferred method for interproximal detection caries, difficulties in reading a radiograph, and what is the cause and misdiagnosis of interproximal caries.

The questions related to the specific aim of the study in Section 2 are as follows:

1. Determining the *presence* of the carious lesion and
2. Ascertaining the *depth* of the carious lesion if present.

For each question, the mean of the correct answers was calculated as indicated in Table 1. Although the students chose BW and digital radiography as their preferred methods for diagnosis, yet the students scored the highest mean of correct *caries detection when using the printed film on paper* method. In other words, there was no significant statistical difference between using a preferred method and achieving a correct diagnosis.

**Figure 1:**
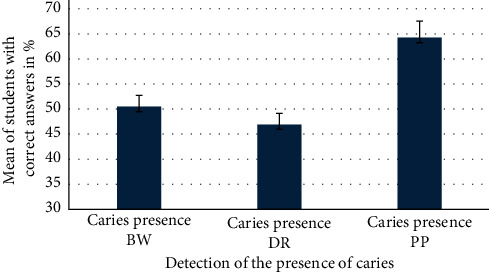
Mean of caries presence with different radiological methods.

At Tygerberg Oral Health Center, the students are familiar with the use of BW radiography and digital radiography, especially the picture archiving communications system. It is mainly used for the detection of interproximal caries. The mean values for the correct caries detection and presence, with the greatest % detected on printed film on paper, are illustrated in Figure 1.

**Figure 2:**
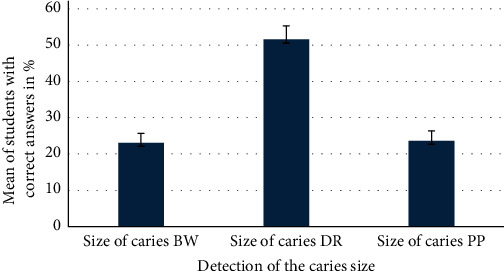
Mean of the caries size with the three different diagnostic methods.

The mean values of the correct caries size estimations were mostly seen on the digital images, as illustrated in Figure 2.

An ANOVA test was carried out for the three different radiographic diagnostic methods as it indicates any statistical differences between the means of three or more independent groups. In other words, it is used for determining the correlation among the variables in statistical analysis. As evident from the *p*-value listed in Table 2 (0.00000881), there is a statistically significant difference for the correct caries detection depending on the radiographic technique used. Within groups, it is sometimes referred to as an error group or error variance. It refers to variations caused by differences within individual groups.

The ANOVA test on the three different diagnostic methods showed that the type of method used affects the size measuring accuracy, where the *P*-value is *p*  < 0.001, confirms a statistically significant difference. This result is shown in (Table 3).

## 5. Discussion

The objectives of this study were partially met even though it was addressed adequately. The 1st objective to determine students' ability to *detect caries* using three different radiographic techniques and to then compare these outcomes statistically was achieved. For the 2nd objective, which was only partially met, as students could not accurately determine the *depth of the lesions* on most radiographic diagnostic methods. Thus, this study acquired a significant amount of information about 5th-year dental students' understanding and usage of caries detection and risk assessment procedures [5, 6]. Previous studies reported similar limitations of students' abilities in cases of interproximal caries detection [39, 40].

This study highlights an important gap in senior dental students' knowledge as they should understand the diagnostic process and the use of detection methods in daily practice as these impact on their diagnostic skills and, ultimately, the treatment of patients. The gap in students' diagnostic knowledge also highlights concerns in the training at a junior level when first exposed to radiographic techniques. It is important to highlight the difference in proximal caries lesion identification but also guiding them to understand the depth of these lesions. The training can be re-empathized regarding the size of lesions in senior years when students have more experience in identifying these as they impact on different treatment approaches.

In addition, the skill and understanding to use available clinical and radiological methods, as in visual tactile sensation in addition to BW radiography, or NILT in addition to BW, each as an adjunct to the other, is very critical [27]. More importantly, having this type of *pairing of diagnostic skills* will allow early detection of carious lesions and thus prevent and control the progression thereof, particularly in their reversible early phases.

For the different radiological diagnostic methods, again, using a *paired approach* when these images are available is equally important. Thus, it was reported that using paired methods of detection radiologically (*BW and printed film on paper* or *BW and digital radiographs*) is more beneficial to correctly detect the presence and size of dental caries, which is why this study addressed the detection of caries using three different radiological methods. In summary, the paired approach includes methods and examples described as *clinical and radiological* or two *different radiological views* [38].

Radwan et al. [43] used a cross-sectional observational study design to assess the methods of caries detection among dental students and dental practitioners in Riyadh and reported that the conventional techniques, such as BW, and invasive methods, such as CBCT of caries detection are common among their participants while scoring low in the advanced diagnostic methods. As a result, Radwan et al. [43] promote minimally invasive approaches and advocate the use and availability of advanced diagnostic methods. In agreement with this, the students of this current study were assessed using conventional diagnostic methods that they are familiar with, BW and digital radiography. In addition to the previous methods, a third one, printed film on paper, was used. The students have scored good results regarding accuracy when detecting the presence of dental caries on all types of radiographic methods, but the detection of the size is reported to be poor on the commonly used ones.

Another cross-sectional study by Nemati et al. [40] reported investigating the accuracy of senior students at Rasht Dental School in detecting interproximal caries. The accuracy of the students in diagnosing the presence of caries was shown to be considerably good in this study. This is in agreement with this current South African study, where students had outstanding recognition of the presence of caries on all three different radiographs. Again, similar to this current study, the Rash Dental School students were only somewhat accurate in identifying the depth of caries, with them understating the depth of caries at the level of the dento-enamel junction. However, contrary to this study by Nemati et al. [40], the 5th-year students in this South African study perfectly detected the depth of the carious lesion on the digital radiographs.

BW radiography is reported as a nonreliable method for early interproximal caries detection, as reported by some researchers [44]. Yet, it has been the most commonly used method globally for the detection of interproximal caries and forms the standard daily diagnostic protocol across private and public dental clinics. It is currently being used by students in teaching hospitals for clinical training worldwide. The current research indicated how unreliable BWs are and that students were more accurate in determining the depth of carious lesions using digital radiographs. In line with this, Radwan et al. [43] encouraged the introduction of advanced radiographic diagnostic methods to clinical practice and training for students and dental practitioners.

Kuhnisch et al. [21] stated that BW radiography can be replaced by NILT due to the similarity in their performance. The reason for NILT being superior is its ability to detect the enamel lesion of interproximal permanent teeth more accurately than BW. This research suggests that NILT may help to minimize the use of BWs. Baltacioglu and Orhan [20], concluded that NILT examination provides a high sensitivity and diagnostic accuracy. It may be used to diagnose caries without using ionizing radiation.

Current literature shows that NILT has a relatively equivalent accuracy to BW for identifying interproximal carious lesions in the permanent teeth. Due to its high accuracy level in detecting interproximal primary caries, NILT is therefore recommended in many special cases only [20]. For example, NILT is more likely to be used for patients who have a high risk of developing caries. Furthermore, it is also favorable in cases when radiation exposure needs to be minimal, such as in pregnant and pediatric patients [45]. However, because of their limited sensitivity, dentists are advised to be attentive in confirming any radiographic findings with a thorough clinical examination for early interproximal lesions [46].

Several other radiographic techniques were developed but never saw use in the clinical space as there were always other systems better than these, for example, intraoral xeroradiography, subtraction radiography, tuned-aperture computed tomography, and machine-intelligence supported systems, to name a few [44]. In dentistry, many other nonradiographic methods have been introduced recently that can guide with diagnosing caries presence and size such as ultrasonography and lasers [47–49]. The limitations of the study centers around the methods students are exposed to clinically, as many institutions still base their teachings on radiographic techniques they are comfortable with rather than newer methods. It is also largely guided by the costs of these techniques; for example, CBCT is only used by specialists in training and not by undergraduate students. This type of mindset has been observed in research from other countries too.

## 6. Conclusion

Senior dental students in this study have shown good caries *detection accuracy* using three different radiographic techniques, especially on printed film on paper. Although the accuracy in detecting the depth/size of carious lesions was poor, there appears to be a reasonable level of understanding and use of diagnostic caries detection and assessment methodologies in restorative treatment planning. It is recommended that dual caries detection methods or what can be called a paired clinical and/or radiographic approach in diagnosis be used that will guide students more effectively.

## Figures and Tables

**Table 1:** Mean of caries presence and size using three different radiological methods.

	Bitewings BW		Digital radiograph DR		Printed paper PP	
	Caries presence BW	Caries size BW	Caries presence DR	Caries size DR	Caries presence PP	Caries size PP
Mean	50.5	23.09091	46.92424	51.5303	64.27273	23.62121
Standard error	2.226588	2.589627	2.194538	3.768612	3.254084	2.767002
Mean of caries presence + size	36.795455		35.007575		49.22727	

Abbreviations: BW, bitewing radiography; DR, digital radiography; PP, printed film on paper.

**Table 2:** One-way ANOVA test for comparison between the presence of the caries with the three different diagnostic methods.

Source of variation	SS	df	MS	*F*	*p*-Value
Between groups	11,075.76768	2	5537.883838	12.36188	*p* < 0.001
Within groups	87,356.21212	195	447.980575	—	—
Total	98,431.9798	197	—	—	—

Abbreviations: df, degree of freedom; *F*, ratio between two mean variables; MS, mean squares; SS, sum of squares.

**Table 3:** One-way ANOVA test for comparison between the size of the caries lesion with the three different diagnostic methods.

Source of variation	SS	df	MS	*F*	*p*-Value
Between groups	34,935.94949	2	17,467.97475	27.79631	*p* < 0.001
Within groups	122,543.4242	195	628.4278166	—	—
Total	157,479.3737	197	—	—	—

Abbreviations: df, degree of freedom; *F*, ratio between two mean variables; MS, mean squares; SS, sum of squares.

## Data Availability

The datasets generated during and/or analyzed during the current study are available from the corresponding authors upon reasonable request. Data generated or analyzed during this study are included in this published article. There is no data associated with this manuscript.
